# Effect of Electroacupuncture on the NTS is modulated primarily by acupuncture point selection and stimulation frequency in normal rats

**DOI:** 10.1186/s12906-017-1690-7

**Published:** 2017-03-31

**Authors:** Jun-Fan Fang, Jun-Ying Du, Xiao-Mei Shao, Jian-Qiao Fang, Zhe Liu

**Affiliations:** Department of Neurobiology & Acupuncture Research, the Third Clinical College, Zhejiang Chinese Medical University, 548 Binwen Road, Binjiang District, Hangzhou, Zhejiang Province 310053 China

**Keywords:** Electroacupuncture, Nucleus of the solitary tract, Frequency, Acupuncture point, Zusanli, Tianshu

## Abstract

**Background:**

The effect of electroacupuncture (EA) is affected by both the acupuncture point selection and the frequency of stimulation. However, little is known regarding acupuncture point and simulation frequency selection. Neuronal activation of the nucleus of the solitary tract (NTS) is one of the important targets of EA for modulating gastrointestinal function. This study investigated the effects of various combinations of EA frequencies and acupuncture points on NTS neurons.

**Methods:**

Rats were randomly divided into normal, 2 Hz EA, 100 Hz EA and the alternate 2/100 Hz EA groups. Then rats in each group were randomly divided into the following two subgroups according to the acupuncture point: ST 36 group and ST 25 group. All the rats underwent electrode implantation surgery. Rats in all EA groups received one treatment with EA (a constant square wave at, 2 Hz,100 Hz or 2/100 Hz frequencies with intensities ranging from 1 to 2 mA), and NTS neuronal activation was recorded before and after EA treatment. Finally, to confirm the effect of EA on the NTS, minimal acupuncture was administered and its effect on NTS was detected.

**Results:**

ST 36 stimulated with 2 Hz EA significantly increased the population of excited NTS neurons and spike frequency. However, ST 36 stimulated with 100 Hz or 2/100 Hz EA produced only a transient effect on the activity of NTS neurons and did not induce any effect on the spike frequency. Furthermore, the excitatory effect of 100 Hz or 2/100 Hz EA on NTS neurons in the ST 36 group was lower than 2 Hz EA at the same point. When applied to ST 25, 2 Hz EA had no significant excitatory effect on NTS neurons or spike frequency. However, 100 Hz EA or 2/100 Hz EA at ST 25 decreased both NTS neuronal excitability and spike frequency. By comparing the effects of different EA combinations, it was shown 2 Hz EA applied to ST 36 had the strongest excitatory effect on NTS neurons, while 100 Hz EA applied to ST 25 had the greatest inhibitory effect. Minimal acupuncture stimulation produced no effect on NTS neurons.

**Conclusion:**

EA’s effects on NTS were mainly affected by the acupuncture point selection, but the frequency of EA also played a role. Different combinations of acupuncture points and frequency selection may lead to different EA effects on NTS neuronal excitability.

## Background

Electroacupuncture (EA), which is a modern way of administering acupuncture, refers to the application of a pulsating electrical current to acupuncture needles for acupuncture point stimulation. Because the stimulatory parameters of EA can be easily and clearly controlled, it has been commonly used in clinical therapy and basic acupuncture research [1–4]. With respect to acupuncture theory, different physical conditions, the selection of different acupuncture points and the quantity of stimulation (frequency of manual acupuncture and EA) all influence the therapeutic effects of acupuncture (including EA) treatment [5]. It is generally believed that the condition of the body mainly determines the terminal effect of EA. However, little is known about the influence of acupuncture points and stimulation method selection on EA effects and their interactions.

EA has been widely used for various gastrointestinal tract (GI) disorders in China and the West [6, 7]. There is a positive relationship between EA regulatory effects on GI motion and activation of the nucleus of the solitary tract (NTS) neurons [8, 9], which is a central nucleus that plays key roles in GI function [10]. It has been demonstrated that the effect of EA on GI was affected by both the acupuncture points and the selected stimulatory frequency [11–14]. However, whether different EA frequency and acupuncture point selections influence NTS neuronal excitability is unknown [9]. Addressing these questions may provide valuable insight towards developing acupuncture mechanisms and aid in understanding the selection principle of acupuncture points and simulation methods.

In this study, we investigated how different combinations of acupuncture points and EA frequency selection influenced the effect of EA on NTS neurons. Two different acupuncture points and 3 EA frequency levels were selected. Zusanli (ST36) and Tianshu (ST25) both belong to the Stomach Meridian of the Foot – Yangming in Chinese acupuncture and are used for treating inconsistent GI diseases [11, 12]. For EA stimulation, 2 Hz, 100 Hz and 2/100 Hz were selected as low, high and alternating frequencies, respectively [14, 13]. In this paper, alternating 2/100 Hz means that the stimulation frequencies of 2 Hz and 100 Hz were applied alternately every 3 s.

## Methods

### Animals and groups

Ninety Sprague-Dawley male rats were obtained from the animal experiment center connected to Zhejiang Chinese Medical University. Rats used in the electro-physiological experiments weighed 280–300 g. The animals were housed with an artificial 12-h light-dark cycle at a controlled temperature (23 ± 1 °C), and relative humidity (70 ± 10%). Distilled water and food were available ad libitum. All animal care, surgery, and handling procedures were approved by the animal experiment center connected to the Zhejiang Chinese Medical University and performed in strict accordance with the National Institutions of Health Guide for the Care and Use of Laboratory Animals (No. 20150117021).

First, 80 rats were randomly divided into the following four groups: (A) a control group receiving normal electrode implantation and no stimulation (*n* = 20); (B) a 2 Hz EA group receiving electrode implantation and 2 Hz EA stimulation (*n* = 20); (C) a 100 Hz EA group receiving electrode implantation and 100 Hz EA stimulation (*n* = 20); and (D) a 2/100 Hz EA group receiving electrode implantation and 2/100 Hz EA stimulation (*n* = 20). All the rats in each group were then randomly divided into the following two subgroups: (i) an ST 36 subgroup receiving EA stimulation at ST 36 and (ii) an ST 25 subgroup receiving EA stimulation at ST 25. Then, 10 rats were randomly divided into the following two groups: (a) a normal group receiving electrode implantation (*n* = 5) and (b) a minimal acupuncture group receiving electrode implantation and minimal acupuncture stimulation (*n* = 5).

### Electrode implantation surgery

Rats were deeply anesthetized with urethane (1 g/kg, i.p.) and transferred to a stereotaxic instrument. A craniotomy was performed for microelectrode array implantation on one side of the brain. According to the atlas of Paxinos and Watson (Edition VI), the NTS was located: 12.8 mm posterior to the bregma, 0.8 mm lateral to the midline, and 5.8 mm ventral to the skull surface. An array of eight stainless steel Teflon-insulated microwires (50 μm) was slowly lowered into the NTS. The microelectrode arrays were secured onto the cranium with stainless steel skull screws and dental cement. Rats were administered penicillin (20,000 U, i.m.) and allowed 7 days to recover [15].

### In vivo electrophysiological recording

The neuronal activities were investigated before and after EA stimulation. During the recording session, rats were allowed to move around freely. The neuronal activities were detected by microwires and passed from the headstage to a pre-amplifier. Single activities were recorded using a 128-channel data acquisition system (Cerebus, Blackrock Microsystems, USA). The neural signals were analog-filtered by the amplifier at cutoff frequencies of 0.3 Hz and 7500 Hz, and digitized with 16-bit resolution at 30,000 Hz using Cerebus Neural signal processors. The digitized signals from each microwire were amplified, digitized, and bandpass filtered from 250 Hz to 5000 Hz. Finally, all signals were saved into a data file for off-line analysis. Spike activities were extracted from the digitized recordings, and individual units were isolated offline using a Plexon Offline Sorter. A signal-unit was defined by homogenous waveforms quantified by sets of waveform parameters clustered in a multidimensional parameter space. The waveform parameters were auto-set by the K-means method, which was built in the software [15].

Ten min of in vivo electrophysiological recording were performed on each rat before EA administered, to characterize the neuronal activity in the NTS and to calculate the average spike firing frequency (baseline). NTS neurons were grouped into the following three types according to their changes: (1) excited, (2) inhibited and (3) no response. For excited neurons, the firing frequency of spikes increased more than 15% over the baseline after EA stimulation. For inhibited neurons, the frequency of spikes was decreased more than 15% from the baseline. All threes type of neurons were counted, and their distribution was calculated and compared to analyze NTS neuronal excitability. Because the frequency of spike firing is normally variable, the rate of change for the spike frequency was calculated and compared to analyze the frequency change.

### EA and minimal acupuncture stimulation

The EA stimulation was administered immediately after the first in vivo electrophysiological recording. During full EA or minimal acupuncture stimulation, rats were loosely immobilized by an assistant’s hand. In the ST 36 subgroup, four stainless steel 0.25-mm-diameter acupuncture needles (Huatuo, Medical supplies factory in Suzhou LLC, China) were inserted at 5 mm depth into the bilateral ST 36 (between the tibia and fibula, 5 mm below the knee) acupuncture points and reference points (1 cm below the ST 36). In the ST 25 subgroup, four subcutaneous needles of the same diameter were inserted at 5 mm depth into the bilateral ST 25 (1 cm beside the release navel) acupuncture points and reference points (1 cm below the ST 25). The two ipsilateral needles were connected to the output terminals of the HANS Acupuncture point Nerve Stimulator (LH-202H, Huawei Co., Ltd., Beijing, China). Electro-stimulation was delivered with the following constant parameters: (1) a constant square wave current output at 2 Hz, 100 Hz or 2/100 Hz (pulse width of 0.5 ms) and (2) intensities ranging from 1 to 2 mA (each intensity for 15 min, totaling 30 min). The minimal acupuncture group received the same acupuncture needle insertion (2 mm in depth) into ST 36 and ST 25 without de qi, and all the needles were linked to the output terminals without electrical stimulation. To eliminate the stress effect, rats in all groups were loosely immobilized by an assistant’s hand similar to the EA and minimal acupuncture group.

### Statistical analyses

The counts for the three types of neurons are represented as count data. The rate of change of the spike frequency is represented as the mean, quartile and standard deviation (SD). All data were analyzed using the Kruskal-Wallis H test followed by the Nemenyi test.

## Results

### The NTS neuronal response to different EA stimulation frequencies at ST 36

**Fig. 1:**
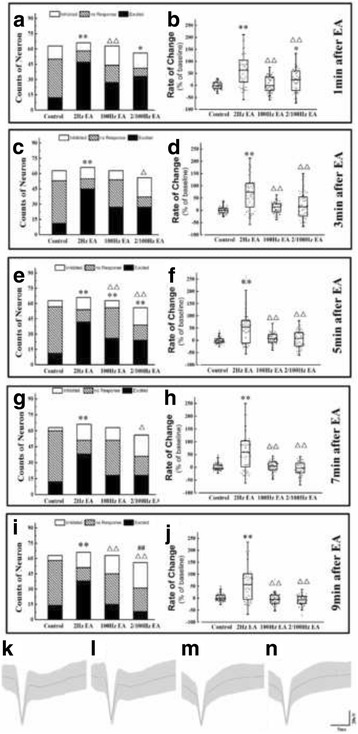
The distribution of three types of neurons and the rate of change for the spike frequency in NTS after EA stimulated at ST 36. The population of three types of neurons of NTS in four groups at 1 min (**a**), 3 min (**c**), 5 min (**e**), 7 min (**g**) and 9 min (**i**) after EA stimulated at ST36. And the rate of change of spike frequency for NTS neurons in four groups at 1 min (**b**), 3 min (**d**), 5 min (**f**), 7 min (**h**) and 9 min (**j**) after EA stimulated at ST36. Results of the number of the three types of neurons are real numbers and the rates of change of spike frequency are mean, quartile and standard deviation. *n* = 5. *P < 0.05, **P < 0.01 versus control group, △*P* < 0.05, △△*P* < 0.01 versus model group, ##*P* < 0.01 versus EA group. Examples show the original traces in the NTS before and after 2 Hz EA (K, M), and examples show original traces in the NTS before and after 100 Hz EA (L, N). The original traces was extracted from 2 to 4 min after recording

Two-hundred-and-forty-eight cingulate neurons from 20 rats were recorded. Sixty-three were from the control group, 66 from the 2 Hz EA group, 63 from the 100 Hz EA group, and 56 from the 2/100 Hz EA group. The number of each type of neuron and the rate of change of the spike frequency are shown in Fig. 1.

In contrast to the control group, EA increased the population of excited NTS neurons to different degrees when EA was applied at ST 36. Only 2 Hz of EA stimulation up-regulated the population of excited NTS neurons during the entire observation time. More than 50% of NTS neurons were excited after 2 Hz EA stimulation (71%, 68%, 64%, 57% and 58%), which was much higher than after 100 Hz EA (46%, 43%, 41%, 29%, and 24%) and 2/100 Hz EA (59%, 48%, 43%, 32%,and 32%) stimulation (Fig. 1a, c, e, g, and i, respectively). However, the population of excited NTS neurons gradually decreased over time in all EA groups. Meanwhile, the spike frequencies of the NTS neurons in the 2 Hz EA group were higher than the 100 Hz and 2/100 Hz EA groups (Fig. 1b, d, f, h, and j). Although 100 Hz and 2/100 Hz EA stimulation at ST 36 had a short-lived increase on the population of excited neurons (<5 min) (Fig. 1a and e) compared to control group, they did not significantly change the spike frequency (Fig. 1b–f). These results indicated that ST 36 is an acupuncture point that tends to increase the excitation of NTS neurons, and its effect on NTS may be partly affected by the stimulation frequency.

### The NTS neuronal response to different EA stimulation frequencies at ST 25

**Fig. 2:**
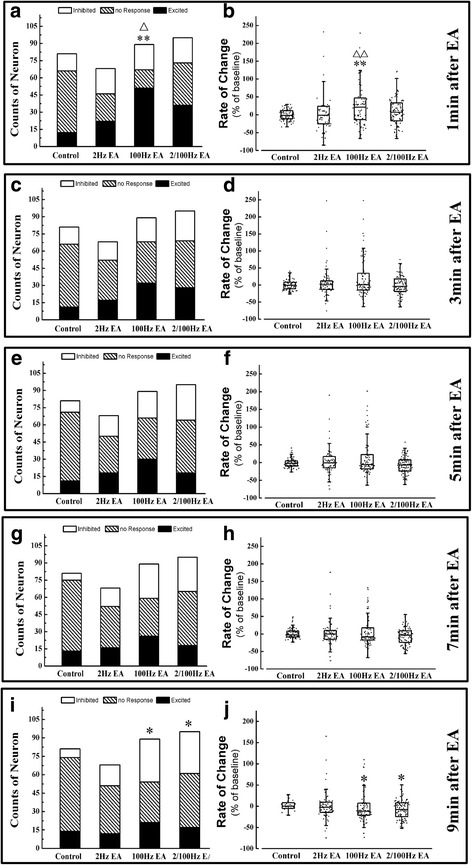
The distribution of three types of neurons and the rate of change for spike frequency change rates in NTS after EA simulated at ST 25. The population of three types of neuron in NTS of four groups at 1 min (**a**), 3 min (**c**), 5 min (**e**), 7 min (**g**) and 9 min (**i**) after EA stimulated at ST 25. And the the rate of change of spike frequency for NTS neurons in four groups at 1 min (**b**), 3 min (**d**), 5 min (**f**), 7 min (**h**) and 9 min (**j**) after EA stimulated at ST 25. Results of the number of three types of neurons are real numbers and the rates of change of spike frequency are mean, quartile and standard deviation. *n* = 5. *P < 0.05, **P < 0.01 versus control group, △*P* < 0.05, △△*P* < 0.01 versus model group

Three-hundred-and-thirty-three cingulate neurons from twenty rats were recorded. Eighty-one were from the control group, 68 from the 2 Hz EA group, 89 from 100 Hz EA group, and 95 from the 2/100 Hz EA group. The results are shown in Fig. 2.

After 100 Hz EA stimulation at ST 25, the population of exited NTS neurons was higher than that of the control rats at 1 min after EA stimulation only (Fig. 2a). Afterwards, the number of no response and inhibited neurons gradually increased and exceeded the excited neurons at 3 and 7 min after EA (Figs. 2c and g). Similarly, the population of no response and inhibited neurons both exceeded the number of excited neurons at 3 and 5 min after administration of 2/100 Hz EA (Figs. 2c and e). Finally, the population of inhibited neurons and the NTS spike frequencies in the 100 Hz and 2/100 Hz EA groups were higher than the control group at 9 min after stimulation (Fig. 2i). Furthermore, the spike frequencies of the NTS neurons in the 100 Hz and 2/100 Hz EA groups were also lower than the control group at the same time (Fig. 2j). On the contrary, 2 Hz of EA stimulation did not cause any changes in NTS excitability when applied at ST 25 (Fig. 2a -j). These results indicated that ST 25 is an acupuncture point that tends to decrease the NTS neuronal excitability, and its effect on NTS was also partly affected by the stimulation frequency.

### The NTS neuronal response to the same EA stimulation frequencies at ST 25 or ST 36

**Fig. 3:**
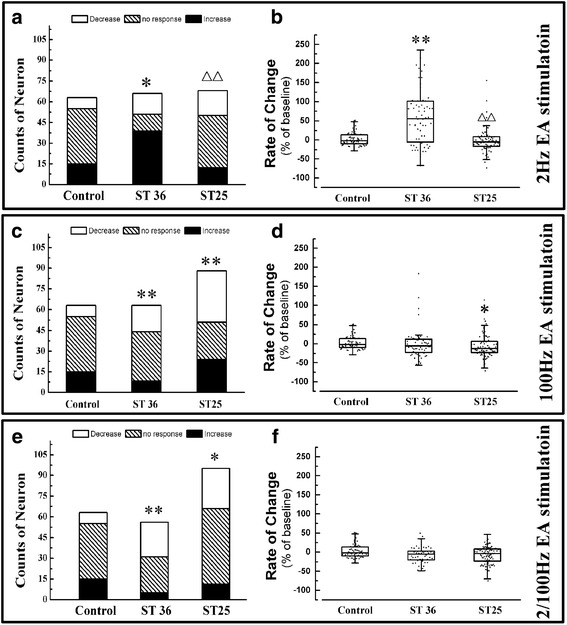
The distribution of three types of neurons and the rate of change for spike frequency in NTS neurons after same EA frequency stimulated at ST 36 or ST 25. The population of three type neuron in NTS at 10 min after 2 Hz (**a**), 100 Hz (**c**) or 2/100 Hz (**e**) EA administrated. The change rates of spike frequency of NTS neurons at 9 min after 2 Hz (**b**), 100 Hz (**d**) or 2/100 Hz (**f**) EA administrated. Results of the counts of three types of neurons are real numbers and the rates of change of spike frequency are mean, quartile and standard deviation. *n* = 5. *P < 0.05, **P < 0.01 versus control group, △*P* < 0.05, △△*P* < 0.01 versus ST 36 group

The interaction between the acupuncture point and stimulation frequency was observed at 10 min after EA stimulation. When ST 36 or ST 25 was stimulated with 2 Hz EA, 197 neurons from fifteen rats were recorded. Sixty-three were from the control group, 66 from the ST 36 group and 68 from the ST 25 group. The population of excited neurons and spike frequencies in the NTS were improved only in EA at ST 36, not at ST 25(Fig. 3a, b).

When ST 36 or ST 25 was stimulated with 100 Hz EA, 214 neurons from fifteen rats were record. Sixty-three were from rats in the control group, 63 from rats in the ST 36 group and 88 from rats in the ST 25 group. The population of excited neurons in the NTS was decreased when EA at ST 36 or ST 25 (Fig. 3c). However, only EA at ST 25 decreased the spike frequency (Fig. 3d).

When ST 36 or ST 25 was stimulated with 2/100 Hz EA, 214 neurons from fifteen rats were recorded. Sixty-three were from the control group, 56 from the ST 36 group and 95 were from the ST 25 group. EA at ST 36 or ST 25 decreased the population of excited neurons in the NTS (Fig. 3e) but not the spike frequency (Fig. 3f).

2 Hz stimulation at ST 36 had the strongest excitatory effect on NTS neurons and 100 Hz stimulated at ST 25 had the strongest inhibitory effect.

### The NTS neuronal response to minimal EA stimulation at both ST 36 and ST25

**Fig. 4:**
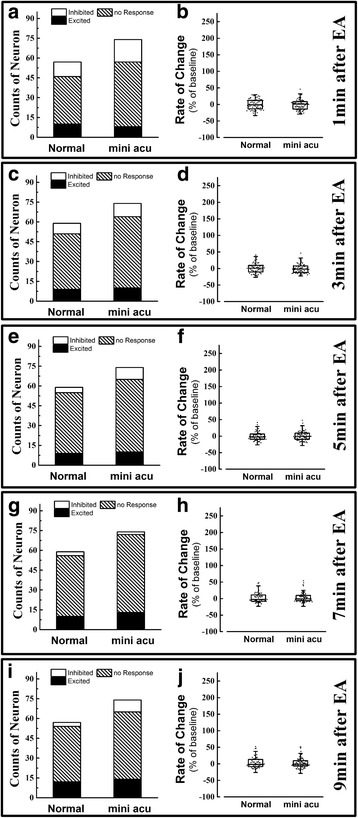
The distribution of three types of neurons and the rate of change for spike frequency change of NTS neurons after minimal acupuncture stimulated at ST 36 and ST 25. The population of three types neuron in NTS at 1 min (**a**), 3 min (**c**), 5 min (**e**), 7 min (**g**) and 9 min (**i**) after minimal acupuncture administered. And the rate of change of spike frequency in NTS neurons at 1 min (**b**), 3 min (**d**), 5 min (**f**), 7 min (**h**) and 9 min (**j**) after minimal acupuncture administered. Results of the number of three types of neurons are real numbers and the rates of change of spike frequency are mean, quartile and standard deviation. *n* = 5. mini acu means minimal acupuncture

Finally, minimal acupuncture was given to confirm the effect of EA on the NTS. In this part, 131 neurons from ten rats were recorded. Fifty-seven were from the normal group and 74 from the minimal EA group. Minimal acupuncture was administered without de qi or any electrical stimulation, so there was minimal effect produced from EA or manual acupuncture. The results indicated that minimal acupuncture stimulation did not influence the population of the three types of neurons and spike frequency when it was delivered at both ST36 and ST25 (Fig. 4a -j).

## Discussion

In the present study, normal rats were used to eliminate the influence of the body condition on the effect of EA as much as possible. All the rats received electrode implantation surgery to eliminate the effects of surgery and anesthesia. We found that the neuronal excitability of the NTS was stable without any stimulation. Different combinations of EA frequencies and acupuncture points led to different effects on NTS neuronal excitability. Furthermore, some combinations do not influence NTS neuronal excitability.

Many clinical studies have shown that acupuncture, including EA, can modulate GI function [16, 17]. A previous study found that somatovisceral reflexes, responsible for regulation of visceral organs, strongly contributed to the effect of EA on GI function [18]. The NTS is the primary center for receiving somatic afferents in the medulla [19]. Interestingly, the NTS is also the parasympathetic center that constitutes the vago-vagal reflex [20, 10]. Different types of afferent vagus fibers transmit various sensory information from the viscera to the NTS [21, 10], which also receives information from higher central nervous system (CNS) that is involved in the regulation of the autonomic nervous system [22]. Then, the NTS integrates this information and sends a signal to regulate the GI tract via efferent vagal nerves [23]. Some studies have demonstrated that EA alters NTS activation via the somatosensory afferent NTS pathway [24, 25]. Here, the results of this study demonstrated that EA, stimulated at either ST 36 or ST 25, induced a significant change in NTS activation. These results are consistent with previous reports [26, 27] and provide support for that the NTS activation is one of the important targets for EA stimulation.

In clinical studies, EA administered at ST 36 improved GI motility and has been used extensively to treat various impaired GI functions and diseases [28–31]. In this study, EA stimulation at ST 36 significantly increased the population of excited neurons in the NTS and the spike frequencies of NTS neurons, which were associated with previous studies [26, 32]. EA stimulation at ST 25 decreased the excitation of the NTS, which was consistent with the suppressive effect of EA on the GI [33, 34]. The opposing effects of EA stimulation were mainly considered to be due to acupuncture point selection. In recent decades, the hypothesis that the location specificity of somatic afferent fibers strongly affected the terminal effect of EA stimulation has been formulated and studied [27, 35, 36]. In this hypothesis, EA or manual acupuncture stimulation at ST 36 may activate vagal nerve fibers via a supra-spinal reflex [26, 27]. Furthermore, EA or manual acupuncture stimulation at ST 25 excited sympathetic efferent nerve fibers via a spinal reflex [27]. We indeed observed the excitatory or inhibitory effects of EA at ST 36 or ST 25 separately on the NTS. However, some results were different from previous studies. For example, a low excitatory effect on the NTS was observed when 100 Hz EA was applied at ST 25. Several reasons may confirm this conjecture. First, anesthetized rats were used in previous studies, and the low excitatory effect of ST 25 on NTS may be modulated by drugs. In contrast, low frequency EA (<30 Hz) or manual acupuncture was used in those studies [37], which were significant different with high frequency or alternating frequency of EA stimulation. Finally, the NTS-rostral ventrolateral medulla (RVLM) pathway also plays an important role in the regulation of EA in the sympathetic nervous system [38]. The underlying mechanism will be further studied in the future. All the above data and results indicate that CNS plays a significant role in EA therapy regardless of where on the body the EA administered.

The frequency of stimulation also affects acupuncture (including EA) treatments similarly as the selection of acupuncture points. A previous study has reported that frequency of twirling manipulation influences the effect of acupuncture at ST 36 [37]. In the present study, the frequency also influences the effects of EA on the NTS. Although both frequencies activated the NTS, the exciting effect of 2 Hz EA on the NTS was greater than that of 100 Hz EA when EA was applied at ST 36. Similarly, 100 Hz EA produced a more pronounced inhibitory effect than 2 Hz EA when EA was applied at ST 25. The effect of 2/100 Hz EA stimulation was close to the average of 2 Hz and 100 Hz EA. In contrast with previous studies, the results of this study showed that both low and high frequencies could affect the supra-spinal CNS, which was significantly different from the previous EA analgesic theory [39]. The integrational effect of the peripheral nervous system or another pathway involved in acupuncture effects may produce these differences; we will continuously focus on the different effects of various EA frequencies and its underlying mechanisms in the future.

Finally, minimal acupuncture was administered at ST 36 and ST 25 simultaneously and did not produce any modulatory effects toward NTS excitation. Because the manual acupuncture was administered without de qi and no electrical stimulation was given, ST 36 and ST 25 would not produce any obvious effect. On the other hand, in this study, data showed that ST 36 and ST 25 produced neuron modulatory effect at different time point. If the minimal acupuncture would produce some effects, it should be observed in the minimal acupuncture data when compared to the normal group. However, there is no significant difference was observed when compared between normal and minimal acupuncture group. Thus, the changes in NTS excitation were mainly induced by EA stimulation in this study.

We also tried to verify the effect of EA on the NTS during EA stimulation. However, the pulsating electrical current from EA interfered with signaling, especially when 100 Hz EA was administered. Nevertheless, excitation of the NTS does not necessarily mean that this activity is producing a therapeutic effect; thus, we will further apply these combinations of EA parameters to GI disease in mammal and study the effects. Finally, some results of the present study, such as the inhibitory effect of high EA frequency at ST 36 and the low excitatory effect of 100 Hz EA at ST 25 were hardly sufficient enough to explain current EA mechanisms. Therefore, we aim to further investigate these results in the future.

## Conclusions

In summary, this study shows that different combinations of acupuncture points and EA stimulation frequencies may lead to different effects on NTS neuronal excitability. This effect is mainly influenced by acupuncture point selection and is also influenced by the EA frequency.

## Abbreviations

CNS: Central nervous system
EA: Electroacupuncture
GI: Gastrointestinal tract
NTS: Nucleus of the solitary tract
